# List of Diseases in June, in the Practice of Dr. Fothergill

**Published:** 1814-07

**Authors:** 


					List of Diseases in June, in the Practice flfDr. Fothergill.
Rubeola
Pcjtussis
Cynanche Tonsillaris 2
Urticaria
Catarrhus
Phthisis Pulraonalis ? * 3
Tussis et Dypsncea ..19
plenvodyne    4
lumbago  2
Khcamatismus A cut us 3
-? Chronic us 5
Cephalalgia  4
Vertigo ? ?.    ?* 2
Paralysis   1
Delirium Ebriosum .. 1
Asthenia  7
Gastrodynia 4
Enterodynia  2
Colica   2
Dyspepsia 3
Pyrosis  1
Hernia   1
Diarrhoea 4
Haeraorrhagia  2
Dysure    I
Ascites    i
Anasarca 3
Hydrothorax ????.. 1
Angina Pectoris ? ?. ? 1
Herpes 2
Porrigo  1
Psora    6

				

